# Beauty in the shadow of neurodegenerative disease: a narrative review on aesthetic experience, neural mechanisms, and therapeutic frontiers

**DOI:** 10.3389/fnhum.2025.1658617

**Published:** 2025-10-09

**Authors:** Andrea Calderone, Rosaria De Luca, Rosalia Calapai, Alessio Mirabile, Angelo Quartarone, Rocco Salvatore Calabrò

**Affiliations:** IRCCS Centro Neurolesi Bonino Pulejo, Messina, Italy

**Keywords:** neuroaesthetics, neurodegenerative diseases, Alzheimer’s disease, Parkinson’s disease, frontotemporal dementia, Huntington’s disease, aesthetic experience, neurorehabilitation

## Abstract

Neuroaesthetics, an emerging field at the intersection of neuroscience, psychology, and the arts, offers new perspectives on the biological and cognitive mechanisms of aesthetic experience. This narrative review explores the convergence of neuroaesthetics and neurodegenerative disorders, focusing on Alzheimer’s disease, Parkinson’s disease, frontotemporal dementia, and Huntington’s disease. Drawing on evidence from neuroimaging, neuropsychology, and clinical studies, we examine how neurodegenerative processes differentially disrupt the neural systems of the “aesthetic triad”: sensory-motor, emotion-valuation, and meaning-knowledge. Such disruptions not only impair patients’ ability to perceive and create art but may also reveal unexpected creative capacities. We discuss the therapeutic potential of arts-based interventions, highlighting the benefits of personalized and technology-driven approaches, including immersive virtual reality and digital art platforms, to enhance neurorehabilitation and psychological wellbeing. The “Michelangelo effect,” where engagement in meaningful aesthetic activities supports learning, motivation, and resilience, exemplifies this translational potential. Our synthesis underscores clinical, neuroscientific, and rehabilitative implications, while noting ongoing challenges such as the need for standardized outcomes and interdisciplinary collaboration. Integrating neuroaesthetic principles into neurorehabilitation may help preserve cognitive and motor functions and enrich quality of life and self-concept in people with neurodegenerative disease. Future research should optimize these approaches to ensure meaningful benefits for patients.

## 1 Introduction

Aesthetic experience, how organisms register, evaluate, and imbue patterned stimuli with significance, arises from entwined biological computations and socio-cultural practices rather than a single, universal essence of “beauty.” In this review, we adopt a plural and reflexive stance: we consider “beauty” a family of experiences shaped by bodies, histories, and contexts, even as we examine shared neural mechanisms with translational relevance for neurodegenerative disease (Chatterjee and Vartanian, 2014; Leder and Nadal, 2014; Masuda et al., 2008). By aligning mechanistic models of prediction, valuation, and meaning with sociocultural diversity, we set the stage for a clinically oriented account of how aesthetic engagement can be leveraged in vulnerable brains. We write from a clinical-neuroscience perspective and do not presume a single global definition of art or beauty; where possible, we triangulate mechanistic accounts with cross-cultural evidence and make our assumptions explicit. This desire to create, experience, and respond to beauty is not simply cultural or symbolic; it is biologically rooted and influences how we think, feel, and behave toward one another (Kim and Lee, 2018). In recent decades, a completely new field of science, called neuroaesthetics, has emerged to address these very questions: What happens in the brain when we experience beauty? What insights can we gain by applying our knowledge to some of the most human health problems? Neuroaesthetics, situated at the intersection of neuroscience, psychology, and the arts, examines how perceivers transform patterned sensory inputs into affectively valued, contextually meaningful experiences across modalities (Magsamen, 2019). It aims to chart the intricate pathways through which sensory input becomes beauty and how, say, art or music and other forms of expression captivate cognitive, emotional, and reward systems in the brain (Cinzia and Vittorio, 2009). Using state-of-the-art technology, including functional magnetic resonance imaging (fMRI), electroencephalography (EEG), and eye tracking, scientists are now unraveling the neural pathways that subtend the appreciation of visual art, music, and literature (Arioli et al., 2025). Converging evidence now couples’ neuroimaging with psychophysiology during art-making itself (e.g., cortisol reduction after visual art-making, EEG alpha changes post-drawing, and HRV modulation across materials), strengthening causal interpretations about reward, regulation, and meaning-making (Kaimal et al., 2016; Belkofer et al., 2014; Haiblum-Itskovitch et al., 2018).

**Figure 1:**
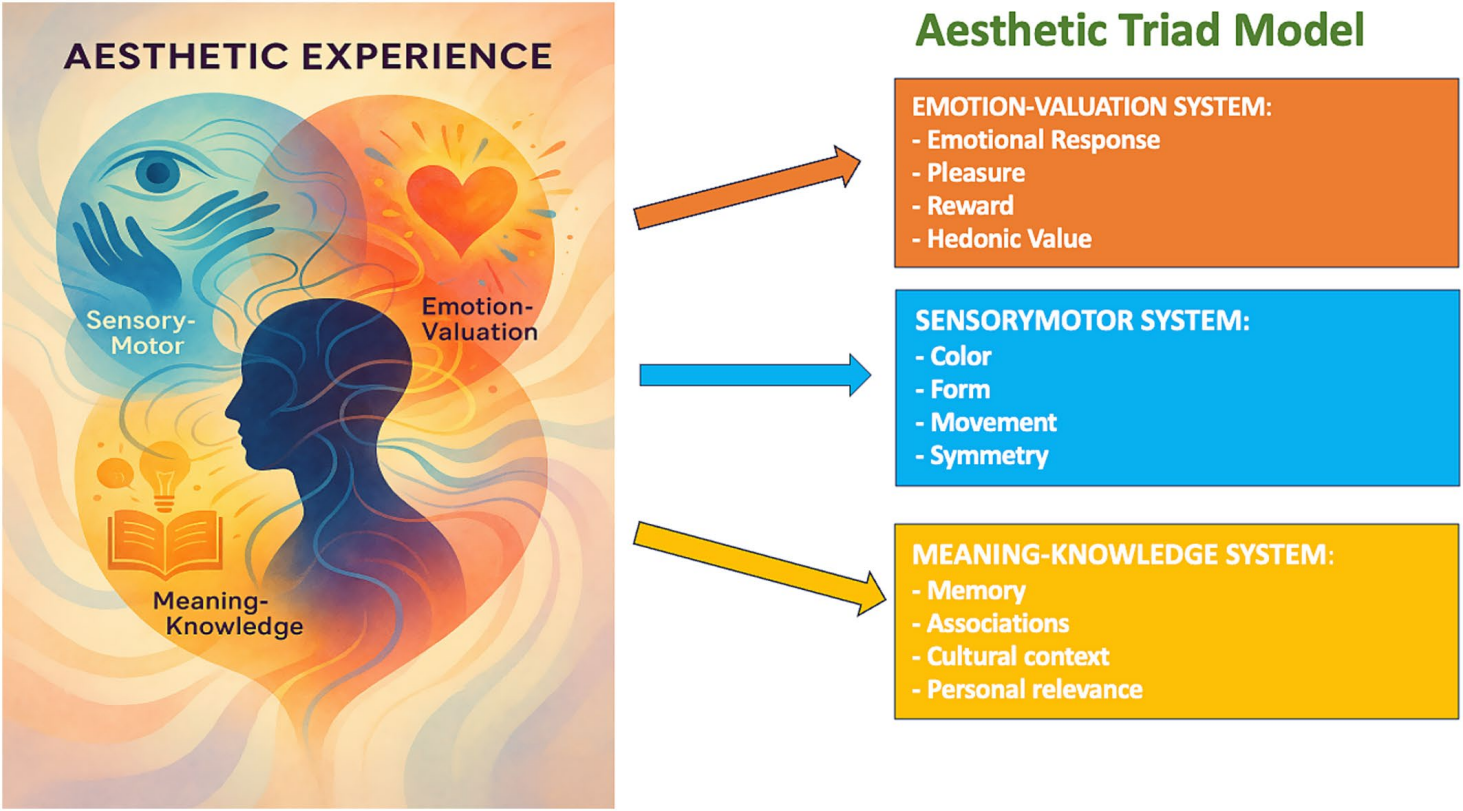
Conceptual map of the aesthetic triad (sensory-motor, emotion-valuation, meaning-knowledge) with example nodes and cross-links (cited in the introduction section).

The “aesthetic triad” model offers an attractive conceptualization suggesting that the experience of aesthetics results from the interplay between three principal neural systems: the sensory-motor system, the emotion-valuation system, and the meaning-knowledge system (Hendriks I. et al., 2019). Complementary appraisal frameworks emphasize multi-stage processing and expectation, underscoring that aesthetic responses are co-constructed by perceivers and contexts rather than fixed properties of stimuli (Chatterjee and Vartanian, 2014; Leder and Nadal, 2014). These mechanisms not only process the physical features of art (symmetry, color, motion) but also assign an emotional value to these feelings, as well as contextual (personal, cultural) meaning (Bara et al., 2022). However, aesthetic experience is by no means a homogenous process. Individual differences abound, shaped by genetic, developmental, cultural, and contextual factors (Germine et al., 2015). Cross-cultural studies show reliable differences in attention and preference, for instance, more context-inclusion in East Asian image composition versus object-focused composition in many Western traditions, highlighting the risk of universalizing any single canon (Masuda et al., 2008; Boduroglu et al., 2009). At the same time, neuroimaging demonstrates that personally meaningful aesthetic appeal recruits’ association networks beyond modality-specific cortices, suggesting a shared biological vocabulary onto which diverse traditions map (Vessel et al., 2019; Bao et al., 2016). Figure 1 illustrates the aesthetic triad (sensory-motor, emotion-valuation, and meaning-knowledge systems) and highlights their cross-links during aesthetic appraisal. Expertise, but also familiarity and even information on the provenance of an artwork, can shape our emotional and neural responses, thus revealing the intricate relationship between subjective appraisal and shared underlying neural systems (Else et al., 2015; Vartanian and Kaufman, 2013). If aesthetic experience is plural and context-shaped yet biologically instantiated, it raises an additional clinical question: what happens when disease limits the capacity of the brain to sense, assign value to, and act upon beauty?

Neurodegenerative diseases, including Alzheimer’s disease (AD), Parkinson’s disease (PD), frontotemporal dementia (FTD), and Huntington’s disease (HD), are some of the deadliest, fastest-spreading, and most prevalent conditions of our age, stealing memory, movement, language, and, ultimately, self. With an aging world population, these are a growing challenge to healthcare, families, and society (Wilson et al., 2023; Kouli et al., 2018; Ulugut and Pijnenburg, 2023; Gadhave et al., 2024; Tesco and Lomoio, 2022; Erkkinen et al., 2018a). The meeting of neuroaesthetics and neurodegenerative disease is a frontier, scientifically and clinically. It encourages us to wonder not only what is lost to disease, but also what might be retained, or even paradoxically amplified. Curiously, some people with frontotemporal dementia experience a surge of creative energy and create labors of uncommon emotional intensity and depth (Shdo et al., 2022). In still other cases, artists with AD or having suffered from a stroke exhibit a change in style, less intricate or more abstract, or a new form of expression, mirroring the plasticity of the artistic mind in the shadow of neural decline (Holmbom Larsen and Londos, 2024; Petcu et al., 2016). Clinically, the therapeutic value of the arts-based intervention is increasingly acknowledged for mobilizing cognitive, emotional, and social profiles of patients living with dementia (Emblad and Mukaetova-Ladinska, 2021). Now music therapy, dance, visual art, and even museum visits are being formally prescribed by doctors for a range of patient needs, including for conditions that are known to be on the decline (Schneider, 2018).

Within this context, the aim of this narrative review is to systematically chart the research on where the fields of neuroaesthetics and neurodegenerative diseases intersect. We aim to elucidate the critical themes and methodological insights that are still in high demand in this realm. To avoid cultural essentialism, we interpret empirical findings through multi-stage appraisal models and cross-cultural evidence that locate aesthetic responses at the intersection of sensory prediction, valuation, and learned meaning. Embarking on this journey, we are aware that neuroaesthetics is a relatively young field and it still has to face several conceptual and methodological challenges. Yet the deep human hunger for beauty, for meaning, and for relationships endures, despite the crumbling of the neurological structures that sustain them. By bridging the worlds of neuroscience, art, and clinical practice, we hope to pave the way for new pathways for research and care. In framing this bridge, we also consider peak aesthetic experiences, commonly reported as chills or frisson, not as epiphenomenal “goosebumps,” but as temporally precise neuromodulatory events that bind reward, attention, and meaning-making and can be purposefully harnessed in vulnerable brains. In what follows, we treat chills as testable mechanisms, rather than metaphors, linking the aesthetic triad to clinically relevant outcomes through measurable changes in learning signals, motivation, and memory consolidation (Schoeller and Perlovsky, 2016; Schoeller et al., 2024). Finally, because aesthetic engagement is being mobilized for vulnerable populations, we explicitly surface issues of ethics, safety, risk, and potential harm. We treat these not as afterthoughts but as design constraints for responsible research and care, spanning consent (including process consent in dementia), privacy of biosignals, cybersickness and overstimulation risks in immersive settings, and equity of access through culturally safe, person-centered protocols (Madary and Metzinger, 2016; Jensen et al., 2024).

## 2 The neuroscientific roots of beauty and artistic perception

Since its introduction by Semir Zeki in 1999, neuroaesthetics has enjoyed a flourishing period of research and development (Zeki et al., 2020; Glennon and Zeki, 2022). Its roots go back much further. At the turn of the last century, 19th-century insight into evolutionary biology and psychology can be traced in particular to Charles Darwin, Gustav Fechner, and Wilhelm Wundt. Starting with the work of these three scholars, constituents for sensory perception and the aesthetic responses it evokes begin to be placed upon a firmer foundation in knowledge; their conceptual breakthroughs spell out how deeply woven into our very nature as an animal (Brattico et al., 2025). At its core, neuroaesthetics aims to untangle the biological underpinnings and neural circuitry that go into the enigmatic and sometimes profoundly personal experience of beauty. The parallelism hypothesis of Zeki, which proposes an inherent similarity between artistic creativity and cerebral organization, is a central theme in this search. Both the artists and the brain, Zeki claims, simplify and stress fundamental visual characteristics in a way that cuts through the innumerable possibilities of visual images, drawing attention to a common underlying urge to understand the visual world (Clarke et al., 2013; Pepperell, 2011; Di Dio et al., 2007; Zeki, 2019). This notion has been the basis of much empirical work in the field of artistic creation and perception, loosening quite radically the separation between art and neuroscience. Methodologically, the growth of neuroaesthetics has coincided with the use of advanced neuroscientific instruments such as fMRI and EEG, which have thus far provided researchers with a unique insight into how aesthetic perceptual experiences engage various parts of the brain (Skov, 2019; Vartanian and Goel, 2004). Especially, a developing literature of meta-analyses converges on the notion of visual aesthetic experience as being subserved by a widespread bilateral neural network, encompassing early occipital visual areas, the ventral visual stream, the fusiform face area, the parahippocampal place area, and the fusiform body area, as well as limbic structures including the amygdala and insula (Jensen et al., 2024). Consistent with this, personally meaningful aesthetic episodes engage default-mode and valuation networks beyond low-level feature detectors, linking appraisal to autobiographical processing (Pelowski et al., 2017). These regions can be differentially engaged with different types of art content (e.g., faces, landscapes, abstract shapes), suggesting that the brain response to art is finely specific to content.

In addition to visual processing, more abstract emotional and cognitive evaluations of beauty involve the medial orbitofrontal cortex, anterior cingulate cortex, and ventral striatum (Boccia et al., 2016). These areas are involved in the reward circuit of the brain and are frequently associated with affective valuation and hedonic pleasure (Rolls, 2023a). Importantly, the orbitofrontal cortex transforms perceptual input and affectively modulates the subjective assessment of beauty, with the release of neurotransmitters, including dopamine in the striatum, being the neuronal correlate of intrinsic reward associated with beauty experiences (Schultz, 2015; Rolls et al., 2020). It is this biochemical substrate that mirrors the one shared by all reward-stimulating behaviors, including those related to eating (Rolls, 2023b) and social bonding, indicating that to experience beauty is not a luxury but an essential aspect of our cognitive functions related to motivation, learning, and memory (Kringelbach, 2005). Beyond this anatomical mapping, peak aesthetic pleasure unfolds dynamically over time: anticipation recruits dorsal striatal circuitry, whereas consummation engages ventral striatum, aligning aesthetic experience with separable phases of dopaminergic signaling (Blood and Zatorre, 2001). Contemporary models therefore interpret chills as moments of precision-weighted prediction error within hierarchical generative models of music and vision, a computation that tags salient patterns and meanings for prioritized encoding and subsequent approach behavior (Schoeller et al., 2024). This time-resolved perspective naturally embeds the triad’s meaning-knowledge system: when a motif returns, a harmonic tension resolves, or a narrative reframes, the co-occurrence of insight and reward is not incidental but mechanistically coupled (Schoeller and Perlovsky, 2016).

### 2.1 The biological basis of aesthetic preference: symmetry and evolution

Research on the human brain’s sensitivity to symmetry, proportionality, and regularity in visual inputs lends credence to the notion that perception and evaluation are linked because symmetry and proportionality cannot be observed before being evaluated (Keefe et al., 2018). While probabilistic preferences for symmetry and proportionality are widely observed, their expression is shaped by cultural learning, genre conventions, and task context; claims of universality are therefore hypotheses to be tested against cross-cultural data. Not only do humans like symmetry in visual stimuli, but non-human primates also prefer such stimuli, implying that a mechanism for detecting and appreciating visual harmony has survived evolution (Sasaki et al., 2005; Little and Griffey, 2020). Cross-cultural and evolutionary psychology research has shown that the preference for specific face and body traits is universal across the human race, implying that aesthetic preference is, to some extent, biologically derived (Little et al., 2011; Fiala et al., 2021). Beyond that, neuroimaging has also found that the insular cortex and anterior cingulate cortex get turned on during moments of particularly strong aesthetic experience, particularly when art provokes deep personal emotional responses (Morita et al., 2014). These areas are involved not only in reward but also in emotion awareness and interoceptive states, highlighting the dual nature of the aesthetic experience as an interoceptive and sensory phenomenon (Brown et al., 2011). Curiously, these networks are more active in people with greater openness to experience or training in the arts, implying a level of plasticity in how the brain attends to beauty (Medford and Critchley, 2010). Recent findings suggest that aesthetic pleasure can also benefit memory and learning, potentially due to its recruiting of attention and emotional arousal, which promote the encoding and consolidation of visual information (Mansueto et al., 2025; Ponzio and Mather, 2014; Belfi et al., 2019).

Yet, this emerging research is not free from conceptual and methodological difficulties. Some have worried about the potential for reductionism, for instance, to reduce complex, culturally embedded experiences with art to just another kind of neural activation or preference rating (Bilsky, 2017). Indeed, because as immensely helpful as the quantitative neuroaesthetic approaches are as indicators and as neural correlates, they should be part and parcel of a general and nuanced understanding of what we can call the contextual, symbolic, and subjective/cognitive dimensions of aesthetic experiences (Pearce et al., 2016). A central challenge for the field, as it continues to grow and evolve, will be to strike the balance between these two extremes, ensuring that the neurobiological perspective not only enriches other, variously informed views of the profound and multilayered nature of art but also enriches them in return. From this vantage, symmetry and proportionality do not merely please because they are regular; they scaffold the predictive models that allow meaningful surprise. Cadential delays, motif recurrences, or perspectival reveals work precisely because the brain has inferred structure and is primed to register precision-weighted violations, a sequence that culminates in chills and durable encoding (Schoeller et al., 2024; Blood and Zatorre, 2001). Methodologically, isolating chills requires convergent markers, time-locked self-report, piloerection sensors, electrodermal activity, and stimuli with controlled expectation profiles to distinguish peak aesthetic response from generic arousal.

## 3 Neurodegenerative disorders and aesthetic experience

As we push beyond the foundational landscape of neuroaesthetics into the lived experience of neurodegenerative disease, we face a paradox: the architectures of meaning within life itself, those that give us the ability to perceive beauty, to connect emotionally to art, and to make, are among the first to be compromised. To avoid over-generalization, we explicitly distinguish disease-general mechanisms from syndrome-specific phenomena and note constraints introduced by sampling frames (e.g., clinic-based, Western-centric) and retrospective caregiver reports. We prioritize convergent evidence from neuroimaging, psychophysiology, and behavior, and we call for preregistered protocols with objective peak-response markers such as piloerection sensors and electrodermal activity to disambiguate specific aesthetic mechanisms from generic arousal (Wassiliwizky et al., 2015; Grewe et al., 2007; Salimpoor et al., 2011). These disorders, in which there is a gradual, selective neuronal decline and widespread propagation of pathology in vulnerable networks, provide a special window upon the most fundamental aspects of human experience (Crutch et al., 2001; Dugger and Dickson, 2017). Clinical syndromes of neurodegenerative diseases, for which their characteristic approaches are developing, are unique representations of points of convergence between impaired long-range networks, emotional responses, and expertise needed to perceive creativity and beauty (Brattico and Varankaitė, 2019). Delving into the study of aesthetic experience in the context of neurodegeneration is more than an academic curiosity; it presents an ideal setting for explaining the distributed facilities and integrative dynamics of aesthetic networks in the brain (Alluri et al., 2012). Each condition, whether AD, PD, frontotemporal dementia, or Huntington’s, is a clinical condition that selectively sabotages neural systems necessary for the perception, appreciation, and creation of art and beauty (Chaudhary et al., 2022). Yet, as pathology assays, the brain is not infrequently an open book of vulnerability and resilience: networks splinter, capacities are dissociated, and, in certain instances, novel capabilities arise (Dugger and Dickson, 2017).

To shed light on these dynamics, we now analyze the influence of the most frequent neurodegenerative diseases on art experience and art production. A cross-cutting theme helps organize this terrain: anhedonia, blunted reward sensitivity, emerges across AD, PD, FTD, and HD and frequently intertwines with late-life depressive syndromes that may foreshadow or accompany neurodegeneration. Conceptualizing aesthetic interventions as tools to restore hedonic tone aligns with evidence that chills recalibrate dopaminergic learning signals, mitigate maladaptive ruminative cognition, and increase approach motivation in depressed patients (Schoeller et al., 2024; Jain et al., 2025). This framing elevates arts-based care from supportive enrichment to mechanistically grounded neuromodulation with testable predictions for memory consolidation, effortful engagement, and mood.

### 3.1 Alzheimer’s disease: the gradual dimming of aesthetic worlds

AD, which has been acknowledged as a prototype of progressive cognitive impairment, also provides a striking example of loss of aesthetic experience. At the earliest stages, AD focuses on the medial temporal lobes, including the hippocampus and entorhinal cortex, before progressively spreading into the temporo-parietal association cortices and frontal lobes. These are, after all, the substrates on which memory, meaning, and affect are organized, central also to the aesthetic triad (Menon and Levitin, 2005). The aesthetic existence of patients with AD is clinically characterized by an insidious and gradual stripping away. Neuroimaging investigations indicate that when patients are exposed to art or music, they have reduced activation of the ventral visual stream and medial orbitofrontal cortex, as well as a reduction of subjective reports of pleasure and an impairment of their ability to make aesthetic judgments (Chaudhary et al., 2022; Menon and Levitin, 2005; Silveri et al., 2015). This appears not just as “not liking art” but as a fracturing of the capability to engage with artistic complexity. Indeed, patients’ responses tend to favor simplicity, formality, and familiar stimuli, compensating little for abstraction or novelty (Leder et al., 2004; Zaidel, 2015). Nonetheless, the emotional power of art can still be reached. A growing literature indicates that aspects of musical memory can remain relatively resilient in AD, particularly recognition of familiar melodies and affective responses to personally salient music, despite severe episodic memory deficits, supporting patient-tailored playlists and caregiver-led music engagement (Cuddy and Duffin, 2005; Jacobsen et al., 2015; Vanstone and Cuddy, 2010). In line with individualized music evidence, personalized listening reduces agitation more reliably than generic ‘relaxation’ music, underscoring the need to calibrate familiarity, tempo, and arousal and to monitor for rare agitation spikes (Park and Pringle Specht, 2009; Belenchia, 2023; Gerdner, 2000). Through observational studies, some patients with early-stage AD can even be reached with music they once knew or even just familiar artwork (Cuddy and Duffin, 2005; Vanstone et al., 2009). Recent papers suggest that although a change in music experience is often reported by patients and carers, music perception can be preserved for well-known music as well as more global affective response to music and known visual art in the early to mid-stages of the disease, despite widespread high-level cognitive deficits that prevent the spontaneous interpretation or analysis of complex artistic material‘s meaning (Arroyo-Anlló et al., 2019). For instance, quantitative data from behavioral and physiological research have supported the notion that, when they listen to personal music, patients with AD express similar affective reactions (e.g., artificial movements, vocalization, or physiological arousal) independently from the presence of verbal recall or recognition deficits (Cuddy et al., 2015; Baird and Samson, 2015; Vink et al., 2013; Särkämö et al., 2013).

The persistent central nervous system awakening response to the negative, noxious events accordingly highlights the relative independence of the cognitive from the emotional processing systems (McKhann et al., 2011). As AD progresses, there is a decrease in generative creativity. Art generated by formerly capable artists is diminished in complexity, nuance, and repetitiousness, which, respectively, are indicative of the loss of executive function and/or semantic memory (Fornazzari, 2005; Miller, 2005). However, procedural memory, the memory for how one performs well-learned activities, can last unusually long, such that an amnesiac can still play a familiar tune even if other abilities are lost (Cavaco et al., 2004). This dissociation highlights the hierarchical arrangement of artistic cognition. Set against this backdrop, selective vulnerability of ventral tegmental area (VTA) dopaminergic neurons in AD models suggests that deficits in reward prediction and motivational drive may emerge before widespread cortical failure (Krashia et al., 2022). Because chills engage VTA-striatal loops and amplify precision-weighted prediction errors, familiar-but-surprising musical or visual sequences may transiently lift hedonic tone and enhance consolidation in mild stages, leveraging preserved affective and procedural channels while limiting cognitive load (Schoeller et al., 2024; Blood and Zatorre, 2001). Practically, this argues for session designs built around personalized playlists with graded tension-release and objective chill markers to titrate difficulty and prevent habituation.

### 3.2 Parkinson’s disease: the disruption of reward and the body’s dialogue with art

PD, a common neurodegenerative movement disorder, is not merely a physiological disorder. The essential pathology, degeneration of dopaminergic neurons in the substantia nigra with resulting depletion of dopamine from basal ganglia and interconnected fronto-striatal circuits, not only affects movement control but also impairs motivation, hedonic tone, and emotional expressiveness (Kalia and Lang, 2015; Weintraub and Mamikonyan, 2019; Radad et al., 2023). Aesthetic participation in PD is thus doubly constrained. On one hand, motor symptoms interfere directly with the production of art: tremor, rigidity, and bradykinesia disrupt the flow of brush, bow, or pen (Thaut and Abiru, 2010; Bologna et al., 2016; Rios-Urrego et al., 2019). On the other hand, non-motor symptoms such as apathy and anhedonia sap the will to engage with beauty at all (Sipos-Lascu et al., 2021; Skorvanek et al., 2015). Neurophysiological studies indicate a muted response in the ventral striatum when individuals with PD are exposed to rewarding or aesthetically pleasing stimuli, a neural correlate of the diminished “reward” response to art (Martínez-Horta et al., 2014; Muhammed et al., 2016). However, the outlook is not entirely negative. Some patients who are treated for pharmacological restoration with dopaminergic tone, particularly dopamine agonists, have paradoxical episodes in which they find themselves flooded with creative energy, which they may manifest compulsively. Reports detail people who, after starting such medications, suddenly develop a newfound interest in an art form or start producing notably more work, sometimes to an obsessional degree (Marafioti et al., 2025; Lhommée et al., 2014; Schwingenschuh et al., 2010). While the condition, which researchers have dubbed “dopaminergic artistic emergence,” has been associated with the neurochemical pathways involved in impulse control disorders, it provides an extraordinary example of the fragile chemical tightrope that underpins the creative urge (Muhammed et al., 2016; Marafioti et al., 2025). Reviews document medication-associated surges in artistic output in a subset of patients, underscoring the narrow therapeutic window between restored motivation and impulse-control risk and the need for systematic monitoring in high-salience interventions (Inzelberg, 2013; Voon et al., 2011). On the phenomenological level, persons with PD commonly report a powerful, if not painful, experience of frustration and loss, as the vivid pictures or music that populate their minds fail to translate themselves faithfully into the gross extent of the motor domain (Gökçal et al., 2017). Many patients describe a continued internal world of artistic vision or music even as the body’s ability to translate those visions into action was progressively eroded (Canesi et al., 2012). If there is a gap between internal creativity and external performance, emotional responses will span from anger to sadness and may result in creative responses such as moving from performance to appreciating passively or finding new alternative, less physically demanding vehicles to come to expression (Spee et al., 2025).

Furthermore, our investigation is in line with others in that, in certain instances, motor deficiency is responsible for limiting creative output, but not beauty perception and emotion, when participants are exposed to familiar melodies, visual art, or poetry (Humphries et al., 2021; Lauring et al., 2019). The fact that the visual pleasure response persevered in the presence of physical limitations is evidence of the multi-level nature of the human brain, on which sensory, emotional, and cognitive networks are deeply entwined but can also at least partly function with great independence. Within this framework, peak aesthetic responses can be used to restore reward learning in sessions that explicitly manipulate expectancy, tempo ramps, rhythmic syncopations, and cadential delays, to drive dorsal-then-ventral striatal phases and counter ventral hyporesponsivity (Blood and Zatorre, 2001). Meta-analytic and clinical evidence confirms high rates of apathy and reward-learning alterations in PD, aligning with blunted ventral striatal responses to incentives and supporting mechanistic rationales for aesthetic protocols that manipulate expectancy and salience (den Brok et al., 2015). Converging evidence from anhedonic depression shows chills-evoked improvements in reward learning and approach motivation, motivating parallel protocols in PD with outcomes spanning apathy, effort-based decision-making, and computational learning metrics (Jain et al., 2025). Because dopaminergic therapy can lower thresholds for impulsive reward seeking, monitoring for impulse-control symptoms should be integrated when designing high-salience aesthetic sessions.

### 3.3 Frontotemporal dementia: the unleashing and Unraveling of creative potential

In the field of the neuroscience of creativity and aesthetics, FTD, comprising a heterogeneous group of disorders involving the frontotemporal lobes bilaterally, profiles as one of the most fascinating clinical entities studied to date (Bang et al., 2015; Seeley et al., 2008). The clinical patterns, behavioral, semantic, and non-fluent aphasias, they disassemble separate components of the aesthetic brain (Rascovsky et al., 2011; Gorno-Tempini et al., 2011). Nowhere is the interrelationship between neural degeneration and creativity more starkly portrayed than in the phenomenon of paradoxical artistic emergence observed in behavioral FTD. Clinical observations and imaging work have characterized artistic behavior in FTD, often with right-hemisphere visuospatial disinhibition and prefrontal atrophy, yielding prolific yet affectively flattened output (Friedberg et al., 2023; Miller and Miller, 2013; Peet et al., 2021; Erkkinen et al., 2018b). Patients who had previously shown little or no interest in the arts can become so absorbed in painting, sculpture, music, and poetry that the artwork itself becomes obsessive, the images vivid or delicately detailed, or the composition compulsively repeated (Rankin et al., 2007). Functional imaging suggests an explanation in the form of disinhibition of posterior right-hemisphere visuospatial regions ‘released’ from the metaplastic control of the atrophic prefrontal cortex. These individuals may stand for hours before the easel with a mixture of creative and pathological compulsion (Ward et al., 2023; Agosta et al., 2012). Their work frequently is not coherent narratively but instead is more emotionally salient or visually complex, marking a move away from goal-oriented construction to a less conscious, more procedural, mode of expression (Antonioni et al., 2023). What caregivers and families often express, with some combination of awe and alarm, is this artistic transformation. And these accounts are evocative precisely because they capture the paradox of FTD, in which the degradation of frontal regulatory circuits unleashes new species of artistic behavior even as it impairs judgment and run-of-the-mill creativity (Miller et al., 1998; Carlsson et al., 2000).

Nonetheless, it is important to point out that this is not a general observation in FTD. Especially the semantic variant, where conceptual knowledge is consistently eroded, distorts the texture of aesthetic experience. People with semantic dementia might begin to find it difficult to recognize familiar objects, faces, or the symbolic content in paintings or sculptures and subsequently disengage with narrative or contextual aspects of art that evoked personal significance (Snowden, 1999). This dissociation, formal exploration with diminished semantic appraisal, motivates stimulus sets that orthogonally manipulate content semantics and low-level structure to parse which components of peak aesthetic responses are preserved across FTD phenotypes (Rouse et al., 2024). What’s more, their interaction with art is primarily concerned with its formal aspects (color, pattern, and texture) isolated from its more profound emotional or representational context. The works that ensued were sometimes successful for their technical brilliance but more often felt emotionally flat or repetitive, the divide between intellectual intent and affective response clearly visible (Drago et al., 2006). This change provides a striking example of the experiential and generative role of art, a product of the bedrock interaction between perceptual, cognitive, and affective networks. The extent to which damage to these networks can both lead to extraordinary bursts of creativity and the utter impoverishment of meaning in FTD indicates the subtle balance that underpins our unique ability to feel and form aesthetic experiences (Ilchovska et al., 2025). Because chills depend on the integration of prediction, violation, and meaning, behavioral-variant FTD may preserve sensory surprise yet distort narrative appraisal, whereas semantic-variant presentations may blunt chills by severing symbolic links that imbue patterns with significance (Schoeller and Perlovsky, 2016). Future studies should pair content-manipulated stimuli with piloerection sensors to parse formal versus semantic contributions to peak responses and to explain why some FTD phenotypes show prolific output that is affectively “flat.”

### 3.4 Huntington’s disease: fractured rhythms of perception and pleasure

HD, an autosomal-dominant neurodegenerative disease involving progressive degeneration of the striatum that eventually includes the cortex, offers a unique problem for the neural circuits that support aesthetic experience (Ross and Tabrizi, 2011; Novak and Tabrizi, 2010). The inexorable spread of HD-mediated neuronal death disrupts not only the sensory-motor networks that are required for artistic production but also the affective-appraisal networks that create emotional reactions to beauty and creativity (Kirkwood et al., 1999; Niccolini and Politis, 2014). Motor symptoms like chorea, dystonia, and severe incoordination can extremely affect the exercise of painting, music playing, or any other artistic expression (Schwartz et al., 2019). But the aesthetic consequences of HD are not limited to motor rendering; they penetrate deep into the patient’s emotional, motivational landscape. Clinically, patients affected by HD often recall a slow reduction of their participation in creative activities and enjoyments. This disconnection is not just a consequence of the effects of physical disability on the mind, but all too frequently it expresses a deeper attrition of desire and pleasure in artistic participation (Atkins et al., 2024). This subjective finding is supported by functional neuroimaging that reveals lower activation of the ventral striatum and orbitofrontal cortex upon reward in patients compared to control subjects (Pardina-Torner et al., 2024; Eidelberg and Surmeier, 2011). These data share some clinical features with the core apathy and anhedonia symptoms (Atkins et al., 2024). Large-scale clinical and imaging syntheses emphasize motivational syndromes as core features of HD and implicate striatal-orbitofrontal circuit compromise during reward processing, supporting short, personalized, high-salience aesthetic blocks with objective engagement markers (Ross et al., 2014; van Duijn et al., 2010). So while a patient does not lose the ability to perceive beauty in the world, in music, art, or nature, often the emotional response is lost that produces the sense of wonder or joy at having the privilege to experience it. On the cognitive front, the very executive deficits that define HD add yet another layer of complexity to life with aesthetics. Deficits in planning, organization, and abstraction could restrict understanding of the complexity of artistic creations, musical narratives, or symbolic content (Schwartz, 2010). This flattening or blunting, an inability to connect deeply to the sources of beauty, for many is one of the most distressing aspects of the disorder, not only because it means pleasure is lost, but also because it means a vital aspect of selfhood is gone (Atkins et al., 2024).

**Figure 2:**
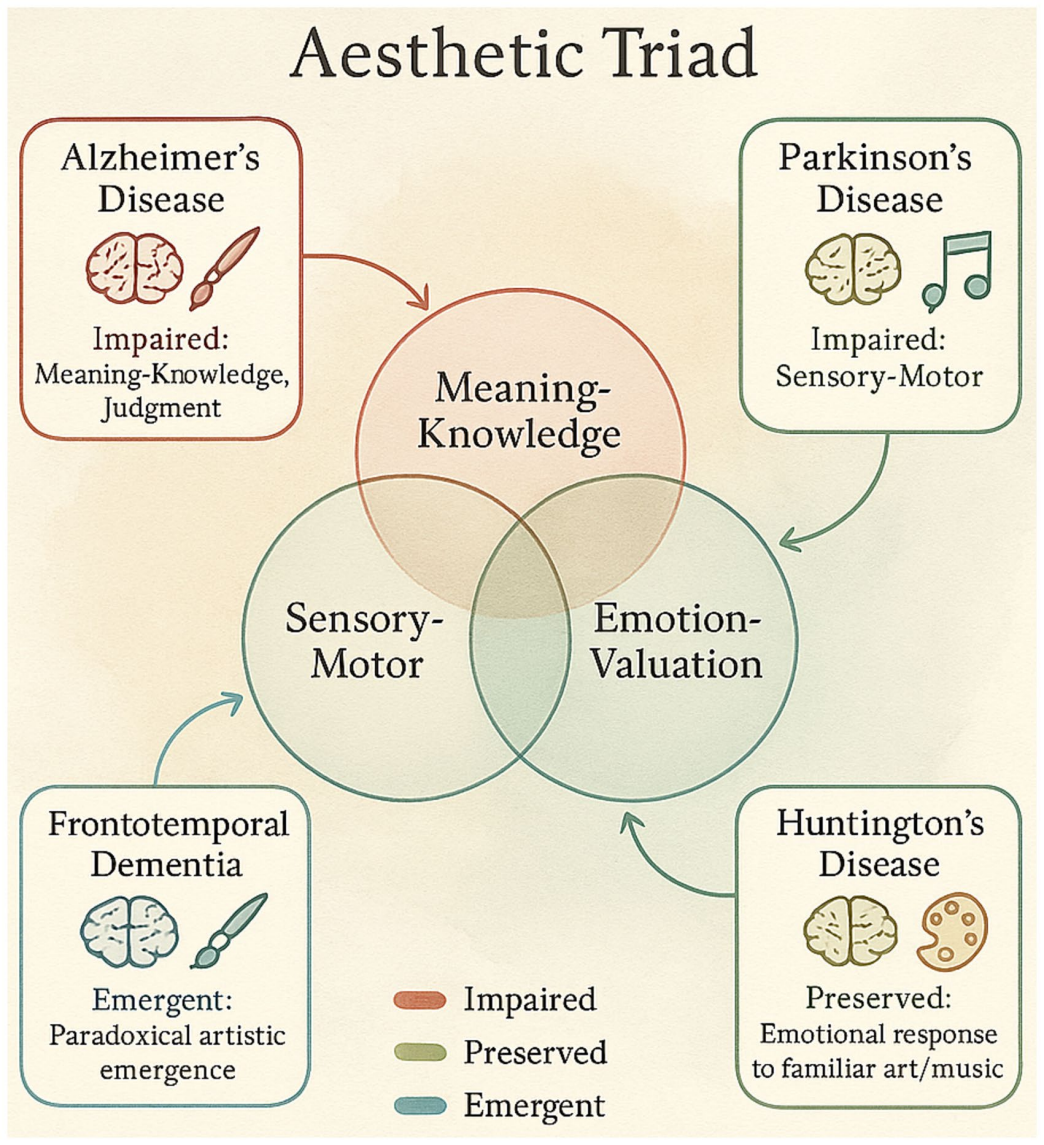
Cross-walk between triad components and syndrome-specific vulnerabilities (AD: medial temporal/valuation; PD: dorsal-ventral striatal phases; FTD: semantic/narrative integration; HD: striatal-orbitofrontal reward) (cited at the end of the 3.4 paragraph).

Moreover, it is important to recognize that people living with HD may perceive and respond to art in many ways. In those who have strong family support and/or a good therapist, simply being around good music, pictures, and aesthetically friendly environments can be a kind of comfort. Such moments may be helpful to intentionally foster from a clinical and research perspective, for even the briefest and subtlest connections to a larger sense of personhood have the potential to support dignity and emotional wellbeing among those with HD (Schwartz et al., 2019). Given striatal degeneration, HD constitutes a stringent test for chills-based strategies: if expectancy-driven sequences still elicit phasic dopaminergic responses, transient gains in engagement and mood may be achievable; however, effects are likely smaller and more fatiguable than in PD/AD, warranting short, highly personalized blocks with objective chill detection and effort-based decision tasks to separate hedonic from motor effects. In light of striatal compromise and fatigue liability, short, personalized aesthetic blocks with embedded chill detection and adverse-event logging are advisable to separate hedonic from motor limits (Lundin et al., 2023; Simón-Vicente et al., 2024). These attempts illustrate the importance of ‘person-centered’ interventions that respect and encourage the remaining modes of expression and communication that may be present even as the disease progresses. For a better understanding of the topic Figure 2 maps the Aesthetic Triad onto syndrome-specific vulnerabilities in neurodegenerative disease (e.g., medial temporal/valuation in AD; dorsal-ventral striatal phases in PD; semantic/narrative integration in FTD; striatal-orbitofrontal reward in HD).

## 4 Painting new pathways: neuroaesthetics at the frontier of neurorehabilitation

The merging of neuroaesthetics and neurorehabilitation is changing how patients with neurodegenerative conditions such as AD, PD, and dementias are treated. Unlike traditional neurorehabilitation, which is generally focused on repetitive, sensory-motor training, the neuroaesthetic approach is based on the acknowledgment of the complexity of the relationship between sensory, emotional, cognitive, and motor functions (Oliva et al., 2023). New frontiers in neuroaesthetics show that observing and producing art activate large networks in the brain related to reward, motivation, memory, and action planning (Nadal and Chatterjee, 2019; Welke et al., 2021). These findings offer potential suggestions on ways that the therapeutic properties of arts and beauty may be utilized to increase participation, optimize neuroplasticity, and protect the psychological wellbeing of those with progressive neurological disease. Importantly, the interventions have been developed to be personalized and scalable to diverse neurodegenerative populations; for example, using new technologies such as immersive virtual reality (VR) and digital art platforms (Vessel et al., 2012; Aldridge and Bethel, 2021; Fu et al., 2023). Converging evidence from public health and rehabilitation syntheses suggests that arts-based and music-based interventions can improve mood, engagement, and some functional outcomes across neurological conditions, while underscoring the need for standardized endpoints and equity-aware implementation (Fancourt and Finn, 2019). VR-enhanced rehabilitation shows small-to-moderate benefits for upper-limb function and activities of daily living in stroke when compared with dose-matched conventional therapy, supporting the scalability of immersive art protocols while motivating rigorous controls for novelty effects (Laver et al., 2017). These results are consistent with an increasing number of reports indicating that art-based or aesthetics-based interventions may prevent the progressive degradation of remaining cognitive-motor capacities in patients with PD and/or AD and can even ameliorate negative conditions (e.g., apathy and depression) often encountered in both disorders (Li Y. et al., 2024; Marco and Redolat, 2023). Positioning anhedonia and its sequelae of apathy and effort aversion as a primary target clarifies why aesthetic protocols can outperform control activities: peak responses appear to deliver precision-weighted dopaminergic signals that support engagement, consolidation, and willingness to exert effort, rather than mere distraction (Schoeller et al., 2024). The authors present neuroaesthetics as an intriguing tool to be used in neurorehabilitation and possibly a fundamental component in the design of a new model of care for such a disease, intended not only to maintain as much as possible the abilities in the more general sense but also to enrich meaning, self-concept, dignity, and quality of life as a whole. The Michelangelo effect is a great example of such a translational trajectory and the mediator of the new, neuroaesthetic-based rehabilitation strategies (Salera et al., 2024). In the next paragraph, we present the Michelangelo effect in its neurobiological context and demonstrate how this concept can be exploited from the perspective of motor and cognitive routines.

### 4.1 The Michelangelo effect: clinical applications of neuroaesthetics in neurorehabilitation

The “Michelangelo effect” refers to the fact that being involved in an aesthetically meaningful activity (such as making or observing art) positively influences skill learning and execution, motivation, and perception of effort (Iosa et al., 2022). Clinical findings, especially based on recent work conducted in stroke populations, emphasize the effectiveness of immersive virtual art therapy protocols based on neuroaesthetic guidelines. For example, De Giorgi et al. (2023), exemplify this strategy with a study where stroke patients engaged in virtual art therapy (VAT), where patients were presented with environments that resembled the ones in which masterpieces of art were painted. Compared with an age-matched group who received standard rehabilitation only, the VAT group made significantly greater gains on upper extremity function, including both Barthel Index (a measure of independence in activities of daily living) and pinch strength (which doubled). Moreover, participants in the virtual art therapy group reported lower rates of muscle fatigue and greater engagement on therapy days, and quantitative findings supported the association between participation in therapy and functional outcomes (De Giorgi et al., 2023). This was supported by preliminary pilot studies (Iosa et al., 2022), suggesting that artistic stimuli presented in a virtual environment would improve not only the kinematics of movements but also the physical demand required by the patients and the task accuracy, as compared with control conditions. To strengthen causal inference, future ‘Michelangelo’ protocols should incorporate preregistered control arms matched on therapist contact and task difficulty, physiological peak-response markers (e.g., piloerection, electrodermal activity), and blinded ratings of movement quality, thereby isolating aesthetic-specific mechanisms from generic attentional arousal (Wassiliwizky et al., 2015; Grewe et al., 2007).

It is worth mentioning that the significance and emotional valence of the aesthetic stimulus seem to play an important role: patients operate with more involvement and better memory, more peacefulness, and less anxiousness when exposed to known or personally meaningful works of art (Kliem et al., 2022). This claim of the Michelangelo effect has been extended beyond painting or even 2D tasks to 3D digital sculpting tasks. Participants sculpting famous works, such as Michelangelo’s David or the Venus de Milo, in VR showed lower jerk and more coordinated bimanual movements relative to those sculpting highly irregular non-artistic shapes, especially during first exposures (Pascucci et al., 2024). While total times for the completed sets of these taxing sculptures were greater, the experience of fatigue was not higher, indicating the role that beauty and aesthetic engagement can play in buffering declining effort over a longer or more challenging task (Iosa et al., 2022). Furthermore, relationships between performance, perceived beauty, and the enjoyment of the task indicate that the art-related motivational “lift” is driven by a combination of neural-sensory priming, emotional arousal, and cognitive involvement (Kirsch and Cross, 2018; Sturm et al., 2023).

**Figure 3:**
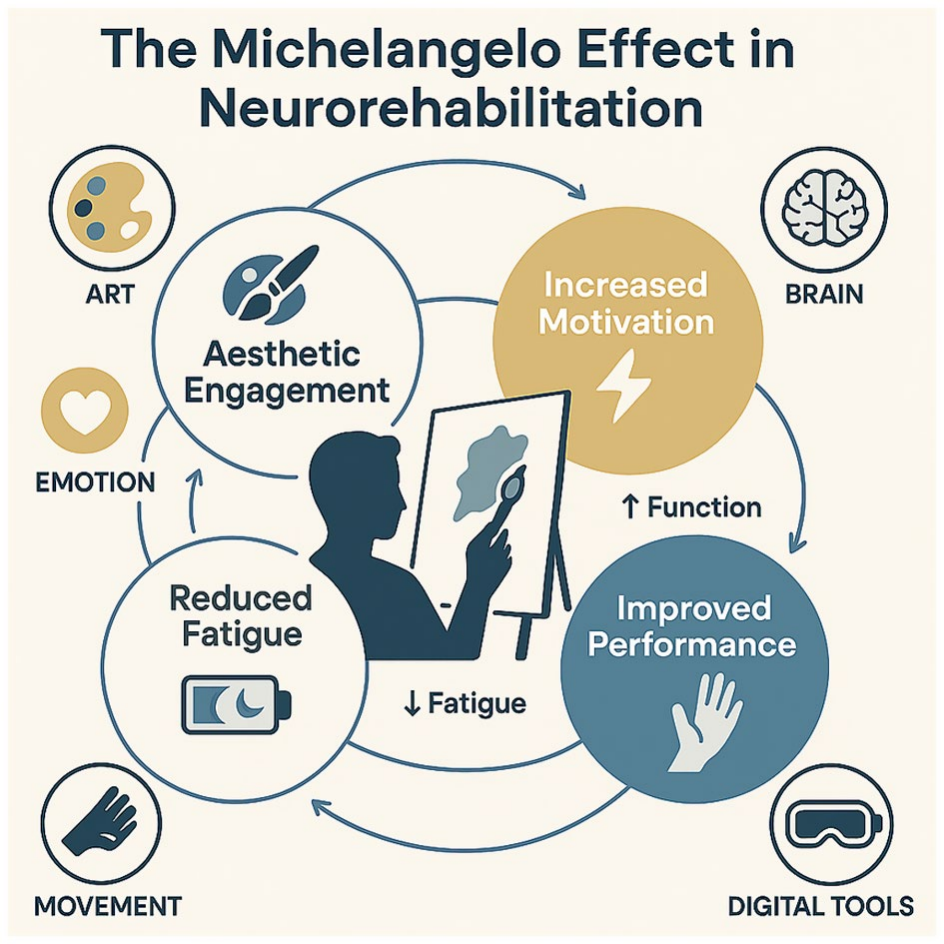
A circular pathway shows how aesthetic engagement (center-left) catalyzes three downstream effects, increased motivation (top-right; contributing to ↑ function), improved performance (bottom-right), and reduced fatigue (bottom-left). Peripheral icons indicate enabling domains (art, emotion, movement, brain) and the role of digital tools (e.g., VR/interactive media) as delivery amplifiers. Curved arrows depict feedback loops whereby motivational lift and performance gains further reinforce engagement over time, while lower perceived effort buffers fatigue during longer or more demanding tasks (cited at the end of the 4.1 paragraph).

Evidence from immersive VR and virtual embodiment shows that body-ownership illusions and agency can modulate motivation and neurorehabilitative outcomes, providing a principled route to personalize aesthetic engagement and effort allocation (Slater and Sanchez-Vives, 2016; Buetler et al., 2022). At the mechanistic level, protocols can also be engineered to elicit chills by shaping expectancy, controlled repetition, harmonic tension, and motif return in order to engage dorsal-then-ventral striatal phases that support learning and pleasure (Blood and Zatorre, 2001). Personalization is now feasible: predictive models can estimate, from brief calibration and person-level features, the probability of a peak response and adapt stimulus parameters in real time to maximize chills while maintaining safety and tolerability (Schoeller et al., 2024). Embedding objective chill markers (e.g., piloerection, electrodermal activity) and preregistered control conditions will help distinguish specific aesthetic mechanisms from novelty or generic arousal. Direct evidence of dopaminergic involvement in peak aesthetic pleasure, via striatal dopamine release during music-evoked chills, supports phase-specific designs that leverage anticipation and tension-release to drive learning signals (Salimpoor et al., 2011). Figure 3 distills the Michelangelo effect: aesthetic engagement, delivered through art and digital tools, increases motivation (functional gains), improves performance, and reduces perceived fatigue via emotion-movement-brain loops. In conclusion, the Michelangelo effect reflects a most promising neural rehabilitation strategy with scientific and clinical relevance in neurorehabilitation. By incorporating aesthetic experience into a patient’s personal therapeutic journey, clinicians may assist patients in addressing their composite needs, promoting neuroplastic facilitation of function and the experience of joy, dignity, and self.

### 4.2 Art therapy and technology: evidence, mechanisms, and practice implications

A rapidly growing literature in art therapy documents psychophysiological benefits that are mechanistically aligned with our framework. In healthy and clinical populations, 45–60 min of visual art-making is associated with significant reductions in salivary cortisol and self-reported stress (Kaimal et al., 2016). Quantitative EEG and meta-analytic syntheses indicate post-drawing shifts in alpha (and in some tasks beta) activity consistent with relaxed alertness and self-regulation (Belkofer et al., 2014). Extending beyond traditional media, a pilot mixed-methods program of virtual drawing reported improvements in affect, creative agency, and wellbeing, suggesting that immersive and multisensory parameters can be tuned to support motivation (Kaimal et al., 2020). Integrative reviews also chart the rapid uptake of digital tools by art therapists (synchronous online work, digital drawing, VR), including practice guidance around competencies, consent, and data governance (Zubala et al., 2021). Finally, museum-based ‘social prescribing’ shows clinically relevant effects on mood and engagement in older adults, bolstering ecological models of cultural participation in care pathways (Thomson et al., 2018). Methodologically, future trials should pair art-making (physical or virtual) with convergent psychophysiology, pre-registered outcomes, and equitable access provisions (device fit, digital literacy) to isolate aesthetic-specific mechanisms from generic arousal (Haiblum-Itskovitch et al., 2018; Czamanski-Cohen and Weihs, 2016).

### 4.3 Ethics, safety, and risk in neuroaesthetic interventions

Ethical and safety considerations are integral to neuroaesthetic protocols. First, consent must be adapted to cognitive vulnerability: a process-consent approach emphasizes ongoing assent, capacity checks, and the right to pause/withdraw without penalty (Hendriks S. et al., 2019). Second, professional codes in art therapy provide concrete guardrails for beneficence, non-maleficence, confidentiality, boundaries, and cultural humility when working with creative content and digital artifacts. Third, immersive technologies warrant explicit risk management. VR can induce cybersickness (nausea, disorientation, and visual discomfort); incidence varies with content and hardware, and protocols should incorporate screening, graduated exposure, rest breaks, seated options, and device-level mitigations (Chang et al., 2020; Ng et al., 2025). Fourth, overstimulation and agitation can occur in a minority of people with dementia during group music/art; personalization and real-time observation reduce risk and support safe cessation when needed (Ridder et al., 2013; Pedersen et al., 2017). Fifth, biosignals used to detect peak responses (e.g., electrodermal activity, piloerection images) constitute sensitive health/biometric data; governance should align with privacy-by-design and emerging ‘neurorights’ discussions addressing mental privacy and cognitive liberty (Ienca and Andorno, 2017). Finally, VR research should follow published ethical recommendations for minimizing harm and reporting adverse events (Madary and Metzinger, 2016). Collectively, these frameworks make explicit how dignity, autonomy, and safety remain primary outcomes alongside efficacy.

## 5 Future perspectives: neuroaesthetics, virtual art, and rehabilitation in the metaverse era

The combination of neuroaesthetics, immersive technologies, and clinical neurorehabilitation is anticipated to usher in a paradigm shift in the treatment of people with neurodegenerative and neurological disorders in the foreseeable future. So far, studies of the Michelangelo effect provide a good level of support for the idea itself, which can be read as aesthetic engagement in the form of making and/or viewing art having the potential to do more than simply lift subjective experiences of patients; it might effectively modulate key rehabilitative outcomes (Salera et al., 2024; Iosa et al., 2022; De Giorgi et al., 2023). The shift from traditional (often monotonous) rehabilitation to engaging, art-based digital environments give rise to new opportunities for improving not only the motor/cognitive abilities but also the mood and motivation of patients. The beginning of VR and its natural growth path into the metaverse is an unprecedented opportunity to build deeply immersive, interactive, and personalizable therapeutic environments. Patients can then actively ‘co-create’ a masterpiece of painting or sculpture in these virtual worlds, as suggested by the study of virtual sculpting protocols in Pascucci et al. (2024), or take a stroll through galleries filled with art stimuli customized to their tastes and experiences. These kinds of environments can mediate between passive and active involvement at once, appreciating art and producing it from a base of embodied, sensorimotor embedding. It is not just a curiosity that exposure to aesthetically significant stimuli can modulate perception of fatigue, enhance bimanual smoothness and movement symmetry, and promote stronger engagement, even under demanding motor demands, it is a clinical opportunity that can be intentionally leveraged (Iosa et al., 2022; Pascucci et al., 2024).

In the future, the most significant leap may well be in the potential for customization and telehealth. The metaverse is set to bring in tele-rehabilitation models under which patients and clinicians can gather, converse, and work together, irrespective of their real-world geography as demonstrated by the study of Calabrò and Morone (2025). This model offers the potential to increase reach, lower costs, and make the best use of therapists’ time while maintaining the human connection that is at the core of good treatment. In addition, the possibility of individualized and immediate adaptation of difficulty level, as well as informative analytics, will support adaptive interventions that are more sensitive and tailored to single patients (Morone et al., 2025). Because aesthetic canons and attentional habits vary across cultures and lifeworlds, content libraries and parameterization (e.g., motif familiarity, narrative framing, tempo envelopes) should be culturally co-designed to avoid imposing narrow taste regimes while maximizing personally meaningful peak responses (Fancourt and Finn, 2019). Another exciting frontier to explore is that of virtual embodiment: the sensation of having and controlling a virtual body or avatar. Studies have shown that this sense of agency and ownership is not just of psychological interest but also a game-changer when it comes to self-perception, motivation, and even the response of neurons (Guy et al., 2023; Li Z. et al., 2024). Seeing virtual hands (or even whole bodies) moving as theirs do in real-time, or even assuming avatars modeled after iconic artists or individuals, often proves to be highly motivating and identity-related (Lougiakis et al., 2020). Embedding validated embodiment techniques, calibrated to patient tolerance and safety, can enhance identification and motivation but necessitates protocols for cyber-sickness screening and graduated exposure (Slater and Sanchez-Vives, 2016). Looking ahead, closed-loop VR should also estimate the instantaneous probability of a chill and adapt predictability, semantic load, and motor challenge in real time, operationalizing precision-weighted learning for rehabilitation. Trials should stratify by anhedonia severity and by suspected prodromal depressive features to test preventative potential suggested by reductions in maladaptive cognition after chills (Schoeller et al., 2024). Finally, because VTA compromise may arise early in AD, phase-specific deployment in preclinical and mild stages deserves priority, with endpoints extending beyond scales to everyday effortful behavior (Krashia et al., 2022).

Nonetheless, the human element cannot be ignored. With these technologies also comes the potential ability to restore not only function but also dignity, meaning, and quality of life as they develop. The arts have always provided a language of hope, expression, and connection, therefore thoughtful incorporation of this into digital neurorehabilitation protocols could be a way to preserve and expand this legacy. The chance to continue creating, perceiving, and interacting with beauty, despite the gradual withering of other abilities, could provide benefits for those living with a progressive neurodegenerative disease that go well beyond what clinical scales can measure. However, there are still some conceptual and methodological challenges. It is important to go beyond novelty effects and to unequivocally differentiate the special contribution of aesthetic involvement, without mere sensory stimulation or technological innovation. Long-term studies will be necessary in order to document persistence of benefits, and parameters used to study patients will have to become standardized and clinically relevant for results to be reproducible and used by other workers. Interdisciplinarity, that is, the involvement of neuroscientists, clinicians, artists, engineers, and patients themselves, will be vital in co-designing interventions that are as meaningful as they are effective.

## 6 Conclusion

The overlap of neuroaesthetics and clinical neuroscience provides an exciting avenue to enhance neurorehabilitation and patient management. What we have seen in the course of this review is that beauty engagement via art, music, or digital environment transcends mere diversion, tapping into neural circuits in support of cognition, motivation, and emotional wellbeing, even as these circuits decline with neurodegeneration. The emergent era of immersive technologies, such as VR and the metaverse, has created an unprecedented momentum for personalized, adaptive, and engaging therapeutic thematics that respect the individuality and creativity of patients. However, future advancement will rely on strong research, agreed-on outcomes, and interdisciplinary teamwork to make certain these interventions are not simply helpful but also accessible. The challenge ahead is to clarify which patients benefit most and to distinguish the unique contribution of aesthetic experience from general novelty or stimulation. The ultimate purpose of the inclusion of neuroaesthetic principles into neurorehabilitation is not only to support function but also to support the conservation of dignity and meaning in the individuals who live with neurological diseases. In closing, treating aesthetic chills as targeted neuromodulatory events that recalibrate dopaminergic prediction and meaning-making reframes arts-based care from enrichment to mechanism-driven intervention, with cross-diagnostic implications for learning, motivation, and prevention. Through making beauty and creativity central to care, the profession will be able to contribute to shaping a world in which healing and wellbeing may become more holistic and more human-centered.
