# Supplementary figures and images for: Beauty in the shadow of neurodegenerative disease: a narrative review on aesthetic experience, neural mechanisms, and therapeutic frontiers
Source: Front Hum Neurosci. 2025 Oct 9;19:1658617. doi: 10.3389/fnhum.2025.1658617 (PMC12546065; doi:10.3389/fnhum.2025.1658617)

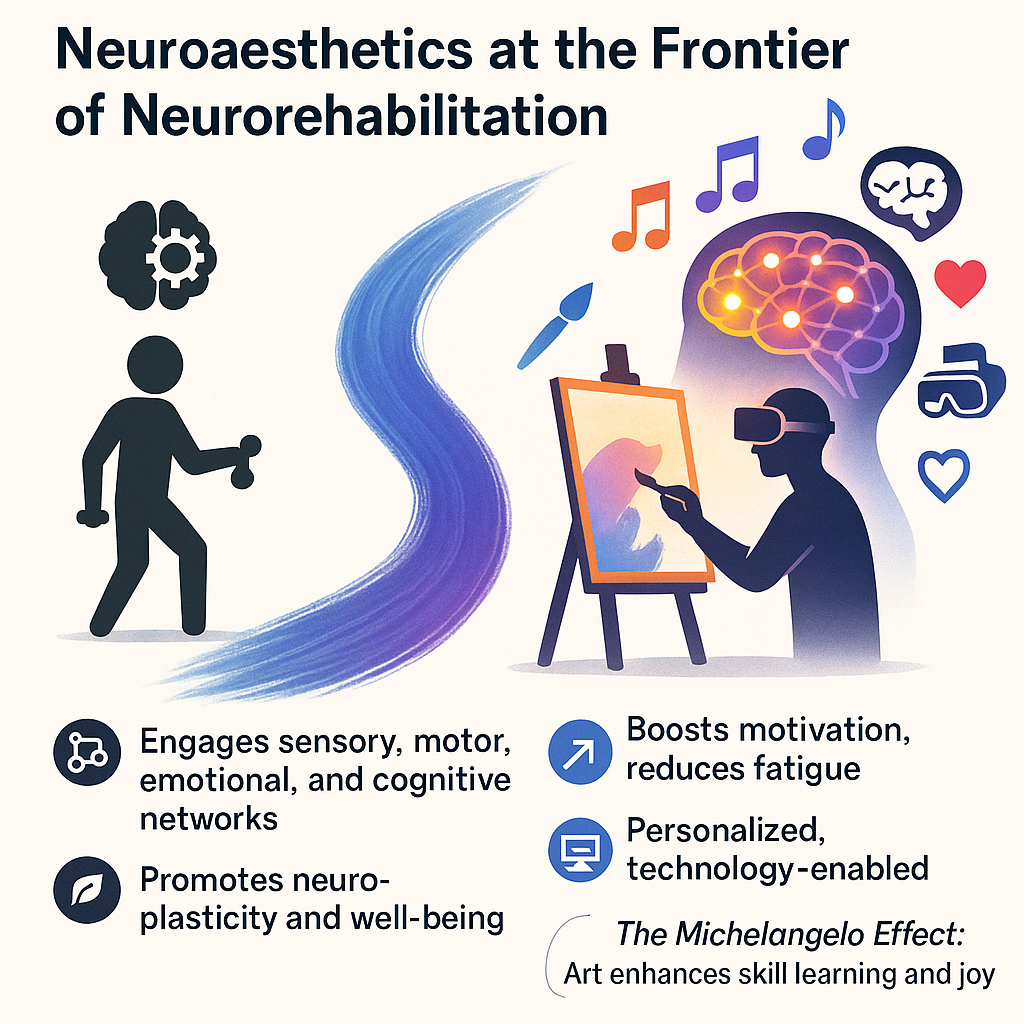

Supplement: Supplementary file 1 [file Image_1.TIFF]
